# Three New Species and a New Record of *Aglaiogyrodactylus* (Monopisthocotyla, Gyrodactylidea, Oogyrodactylidae) from *Hisonotus* spp. (Siluriformes, Loricariidae) in Brazil

**DOI:** 10.1007/s11230-026-10293-9

**Published:** 2026-08-03

**Authors:** Sabrina Dalmas-Branchi, Tainã G. Da Silva, Marcus V. Domingues, Lizandra J. Robe, Rogério T. Vianna

**Affiliations:** 1 Laboratory of Systematics and Coevolution, Institute of Coastal Studies, Federal University of Pará, Bragança, Brazil; 2 Graduate Program in Environmental Biology, Federal University of Pará, Bragança, Brazil; 3 Laboratory of Biology of Aquatic Organism Parasites, Institute of Biological Sciences, Federal University of Rio Grande, Rio Grande, Brazil; 4 Graduate Program in Biology of Continental Aquatic Environments, Institute of Biological Sciences, Federal University of Rio Grande, Rio Grande, Brazil; 5 Department of Ecology and Evolution, Federal University of Santa Maria, Santa Maria, Brazil

## Abstract

*Aglaiogyrodactylus* Kritsky, Vianna & Boeger, 2007 comprises ectoparasitic metazoans recorded mainly on the body surface of loricariid fishes. Although the genus currently includes only eight species, its actual diversity remains largely underestimated. In this study, three new species of *Aglaiogyrodactylus* collected from specimens of *Hisonotus* spp. in Brazil are described, and a new locality record of *Aglaiogyrodactylus pedunculatus* Kritsky, Vianna & Boeger, 2007 is reported. The descriptions are based on diagnostic morphological characteristics, with the male copulatory organ as the main distinguishing structure. *Aglaiogyrodactylus matildeae* **n. sp.** presents an accessory piece composed of two branches: a sinistral spatulated branch and a dextral tubular branch. *Aglaiogyrodactylus nonnae* **n. sp.** possesses an accessory piece divided into three branches: two sinistral spatulated branches that serve as a guide for the male copulatory organ and a bifurcated dextral branch derived from an annular structure. *Aglaiogyrodactylus marinae* **n. sp.** presents an accessory piece formed by three branches, one spatulated sinistral branch with a semicircular end, and dextral branches with distal ends having inconspicuous digitiform projections. These descriptions expand the taxonomic knowledge of *Aglaiogyrodactylus* and document the occurrence of new lineages associated with *Hisonotus* spp. in southern Brazil.

**Supplementary Information:**

The online version contains supplementary material available at https://doi.org/10.1007/s11230-026-10293-9.

## Introduction

Siluriforms account for approximately 42% of the continental fish diversity in South America, with Loricariidae Bonaparte, representing the most species-rich lineage. Currently, Loricariidae comprises seven subfamilies, 130 genera, and 1,069 valid species (Albert et al., 2020; Fricke et al., 2025). Despite their remarkable diversity, ecological importance, and relevance to the international ornamental fish trade, only 4% of loricariid species have been investigated for metazoan parasites (Branches et al., 2021).

Among the metazoan parasites of loricariids, those belonging to the Oogyrodactylidae Harris, 1983 (Platyhelminthes, Monopisthocotyla) are particularly noteworthy (Harris, 1983; Kritsky et al., 2007; Da Silva et al., 2025). Oogyrodactylids occur exclusively in the Neotropical Region, with records restricted to South America. Most species have been reported from loricariid catfishes (Boeger et al., 2020), whereas only a few records are known from pimelodid hosts, including *Phanerothecium caballeroi* Kritsky & Thatcher, 1977 from *Zungaro zungaro* (Humboldt), and *Phanerothecioides agostinhoi* Kritsky, Vianna, & Boeger, 2007 from *Pseudoplatystoma fasciatum* (Linnaeus). These occurrences may be considered incidental since sexually mature individuals have not been found in these host species (Kritsky et al., 2007). Currently, Oogyrodactylidae comprises 25 valid species distributed among eight genera: *Aglaiogyrodactylus* Kritsky, Vianna & Boeger, 2007 (8 species), *Atopogyrodactylus* Kritsky, Boeger & Patella, 2020 (1 species), *Hyperopletes* Boeger, Kritsky & Belmont-Jégu, 1994 (1 species), *Nothogyrodactylus* Kritsky & Boeger, 1991 (3 species), *Onychogyrodactylus* Kritsky, Vianna & Boeger, 2007 (2 species), *Oogyrodactylus* Harris, 1983 (1 species), *Phanerothecioides* Kritsky, Vianna & Boeger, 2007 (1 species), *Phanerothecium* Kritsky & Thatcher, 1977 (8 species) (Table 1).

**Table 1:** Valid species of Oogyrodactylidae Harris, 1983, their recorded host subfamilies and families, and the respective type localities (state and country)

Species of Oogyrodactylidae	Host subfamily and family	Locality	References
***Aglaiogyrodactylus* Kritsky, Vianna & Boeger, 2007**			
*A. calamus* Kritsky, Vianna & Boeger, 2007	Hypoptopomatinae (L)	Paraná, BR	Kritsky et al. (2007) Patella et al. (2017)
*A. conei* Kritsky, Vianna & Boeger, 2007	Hypoptopomatinae (L)	Paraná, BR	Kritsky et al. (2007)
*A. ctenistus* Kritsky, Vianna & Boeger, 2007	Hypoptopomatinae (L) Hypostominae (L)	Paraná, BR	Kritsky et al. (2007) Patella et al. (2017)
*A. forficulatus* Kritsky, Vianna & Boeger, 2007	Hypoptopomatinae (L)	Paraná, BR	Kritsky et al. (2007) Bachmann et al. (2016) Patella et al. (2017)
*A. forficuloides* Kritsky, Vianna & Boeger, 2007	Hypoptopomatinae (L)	Paraná, BR	Kritsky et al. (2007) Patella et al. (2017)
*A. guttus* Kritsky, Vianna & Boeger, 2007	Hypoptopomatinae (L)	Paraná, BR	Kritsky et al. (2007) Patella et al. (2017)
*A. pedunculatus* Kritsky, Vianna & Boeger, 2007	Hypoptopomatinae (L) Hypostominae (L) Loricariinae (L)	Paraná, BR	Kritsky et al. (2007) Patella et al. (2017)
*A. salebrosus* Kritsky, Vianna & Boeger, 2007	Hypoptopomatinae (L)	Paraná, BR	Kritsky et al. (2007)
*A. marinae* **n. sp.**	Hypoptopomatinae (L)	Rio Grande do Sul, BR	**This study**
*A. matildeae* **n. sp.**	Hypoptopomatinae (L)	Rio Grande do Sul, BR	**This study**
*A. nonnae* **n. sp.**	Hypoptopomatinae (L)	Rio Grande do Sul, BR	**This study**
***Atopogyrodactylus* Kritsky, Boeger & Patella, 2020**			
*A. praecipuus* Kritsky, Boeger & Patella, 2020	Hypostominae (L)	Rondônia, BR	Kritsky et al. (2020)
***Hyperopletes* Boeger, Kritsky & Belmont-Jégu, 1994**			
*H. malmbergi* Boeger, Kritsky & Belmont-Jégu, 1994	Loricariinae (L)	Amazonas, BR	Boeger et al. (1994)
***Nothogyrodactylus*** Kritsky & Boeger, 1991			
*N. amazonicus* Kritsky & Boeger, 1991	Hypostominae (L)	Amazonas, BR	Kritsky & Boeger (1991)
*N. clavatus* Kritsky & Boeger, 1991	Hypostominae (L)	Amazonas, BR	Kritsky & Boeger (1991)
*N. plaesiophallus* Kritsky & Boeger, 1991	Hypostominae (L)	Amazonas, BR	Kritsky & Boeger (1991)
***Onychogyrodactylus* Kritsky, Vianna & Boeger, 2007**			
*O. hydaticus* Kritsky, Vianna & Boeger, 2007	Hypoptopomatinae (L) Hypostominae (L)	Paraná, BR	Kritsky et al. (2007)
*O. sudis* Kritsky, Vianna & Boeger, 2007	Hypostominae (L)	Paraná, BR	Kritsky et al. (2007)
***Oogyrodactylus*** Harris 1983			
*O. farlowellae* Harris, 1983	Loricariinae (L)	Peruvian Amazon, PE	Harris (1983)
***Phanerothecioides* Kritsky, Vianna, & Boeger, 2007**			
*P. agostinhoi* Kritsky, Vianna & Boeger, 2007	Hypostominae (L) Pimelodidae	Tocantins, BR	Kritsky et al. (2007)
***Phanerothecium*** Kritsky & Thatcher, 1977			
*P. acutum* Da Silva, Aguiar, Ebert & Silva, 2025	Hypostominae (L)	São Paulo, BR	Da Silva et al. (2025)
*P. caballeroi* Kritsky & Thatcher, 1977	Pimelodidae	Cuaca, CO	Kritsky & Thatcher (1977)
*P. deiropedeum* Kritsky, Vianna & Boeger, 2007	Hypostominae (L)	Paraná, BR	Kritsky et al. (2007)
*P. harrisi* Kritsky & Boeger 1991	Hypostominae (L)	Amazonas, BR	Kritsky & Boeger (1991) Kritsky et al. (2007)
*P. macrosomum* Vianna, Pelegrini, Vieira, Azevedo & Abdallah, 2022	Hypostominae (L)	São Paulo, BR	Vianna et al. (2022)
*P. spinatum* Boeger, Kritsky & Belmont-Jegu, 1994	Hypostominae (L)	Rio de Janeiro, BR	Boeger et al. (1994)
*P. spinulatum* Kritsky, Vianna & Boeger, 2007	Hypostominae (L)	São Paulo, BR	Kritsky et al. (2007)
*P. spinatoides* Kritsky, Vianna & Boeger, 2007	Hypostominae (L)	Tocantins, BR	Kritsky et al. (2007)

L= Loricariidae; BR= Brazil; PE= Peru; CO= Colombia

*Aglaiogyrodactylus* and *Phanerothecium* exhibit the highest species diversity within the Oogyrodactylidae (Kritsky et al., 2007; Da Silva et al., 2025). Representatives of *Aglaiogyrodactylus* have been recorded from loricariid fishes belonging to Hypoptopomatinae Eigenmann & Eigenmann, Loricariinae Rafinesque, and Neoplecostominae Regan, predominantly in southern Brazil. (Table 1) (Kritsky et al., 2007; Patella et al., 2017). Members of this genus are characterized by having the accessory piece of the male copulatory complex confined within the copulatory sac. This structure supports the copulatory sac and serves as a guide for the male copulatory organ during copulation (Kritsky et al., 2007).

In this study, we describe three new species of *Aglaiogyrodactylus* and a new locality record of *A. pedunculatus* Kritsky, Vianna & Boeger, 2007, from *Hisonotus* spp. hosts from southern Brazil.

## Materials and methods

A total of 129 specimens of *Hisonotus* spp. were collected using a sieve and dip net in tributary streams of the Patos Lagoon, Rio Grande do Sul, Brazil, during 2018 and 2019. The fish were anesthetized with an alcoholic solution of Eugenol (110 mg L⁻^1^), according to Braz et al. (2017), and subsequently euthanized with Eugenol (400 mg L⁻^1^). Following this procedure, the specimens were individually placed in vials containing hot water (70 °C) and vigorously shaken. Parasites and hosts were then preserved in 96% ethanol (Kritsky & Stockwell, 2005).

The oogyrodactylids were separated using a stereomicroscope and the aid of needles. Each specimen was individually mounted in Hoyer's medium or Gray & Wess medium, or stained with Gomori's trichrome and mounted in Canada balsam (Humason, 1979; Boeger & Vianna, 2006) for subsequent morphological characterization. Diagnostic characters and morphological terminology followed Kritsky et al. (2007). Drawings were prepared using an Olympus BX51 optical microscope equipped with a bright-field camera, and the photographs were taken using an Olympus CX41 microscope equipped with phase contrast and an Olympus DP73 camera. Measurements were obtained from photographs using ImageJ 1.52v (Schneider et al., 2012), and are reported in micrometers (μm), as the mean followed by the minimum and maximum values and the number of measurements taken (n) in parentheses. Type specimens and voucher specimens examined in this study were deposited in the Helminthological Collection of the Instituto Oswaldo Cruz (CHIOC), Rio de Janeiro, Rio de Janeiro State, Brazil. Ecological descriptors, including prevalence and mean infection intensity, were estimated according to Bush et al. (1997).

## Results

The present study describes three new species of *Aglaiogyrodactylus* and reports a new locality record for *Aglaiogyrodactylus pedunculatus* Kritsky, Vianna & Boeger, 2007. All newly described species conform to the generic diagnosis of *Aglaiogyrodactylus* proposed by Kritsky et al. (2007) and are therefore assigned to this genus.

Class Monopisthocotyla Brabec, Salomaki, Kolísko, Scholz & Kuchta, 2023

Order Gyrodactylidea Bychowsky, 1937

Oogyrodactylidae Harris, 1983

*Aglaiogyrodactylus* Kritsky, Vianna & Boeger, 2007

**Figure 1.**
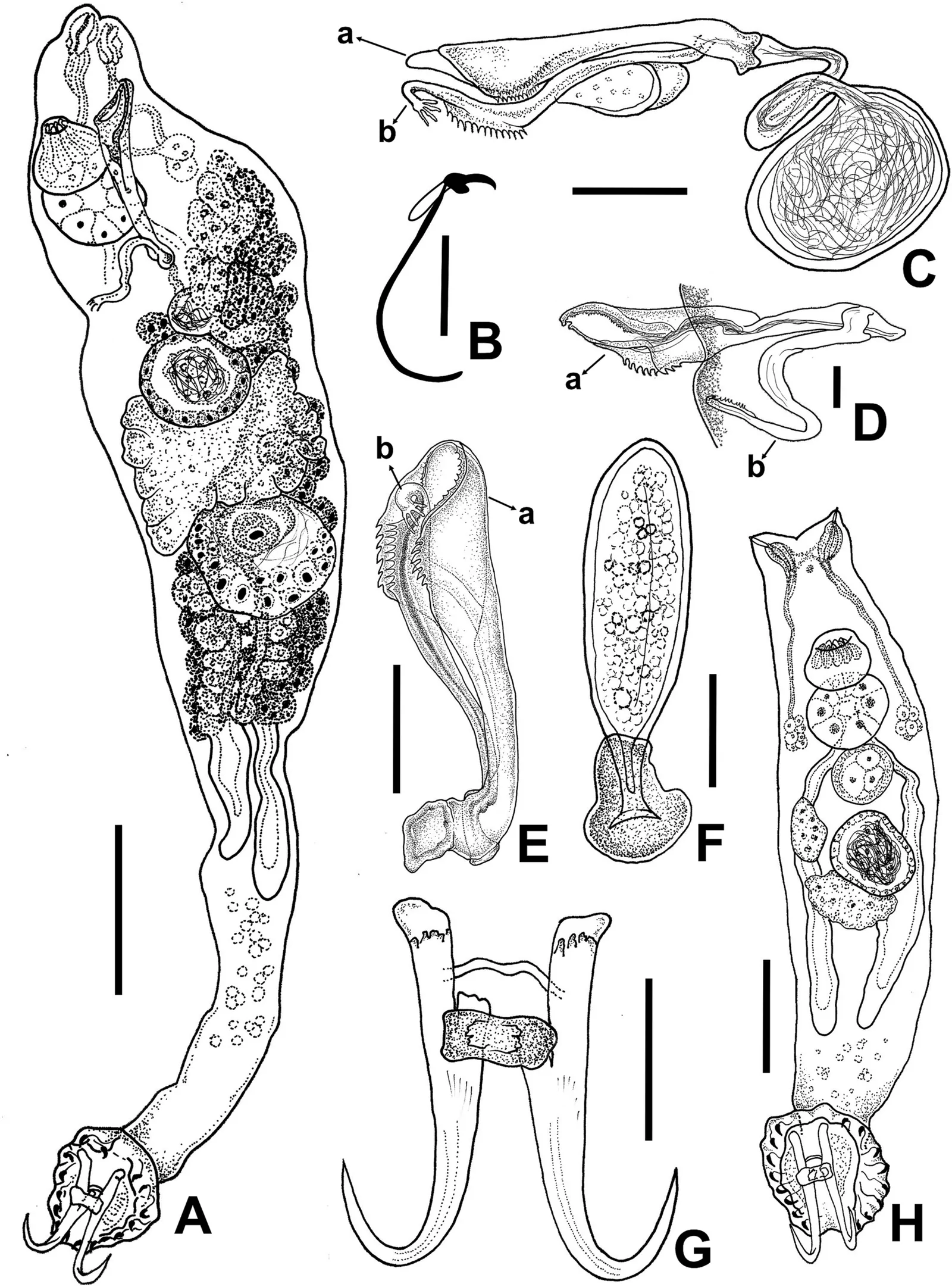
*Aglaiogyrodactylus matildeae* **n. sp.** **A**: Adult. **B**: Hook. **C, D, E**: Male copulatory complex; **a** – sinistral branch, **b** – dextral branch. **F**: Egg. **G**: Anchor and bar complex. **H**: Juvenile. Scale bar: A = 200 µm; C, D, and F = 10 µm; B, E, and G = 25 µm; H = 50 µm.

***Aglaiogyrodactylus matildeae* n. sp.** (Fig. 1, S1. A, B)

**Type host:** *Hisonotus laevior* Cope (Loricariidae).

**Site of infestation:** Body surface.

**Prevalence:** 53.4% of 73 hosts examined.

**Mean infection intensity:** 8.2 parasites per host.

**Type locality:** Arroio das Cabeças Stream (32°04'36.4"S, 52°15'08.9"W), Vila da Quinta, Rio Grande, Rio Grande do Sul, Brazil, January 2019.

**Specimens deposited:** Holotype, CHIOC 40901; 14 paratypes, CHIOC 40902 – 40910, 41001 – 41005.

**Etymology:** This species is named in honor of Matilde Romeu Tubino, mother of the senior author, as a way to immortalize her legacy and influence on the author's life and career.

**Zoobank:** urn:lsid:zoobank.org:act:C7C15B21-E365-40B7-B66E-D6D4E694E317

**Measurements:** Body 1,035 (897–1,201; n=4) length, 205 (189–219; n=4) width; posterior pharyngeal bulb 44 (42–48; n=5) width, anterior pharyngeal bulb 31 (23–34; n=5) width. Accessory sinistral piece 93 (86–97; n=4), accessory dextral piece 80 (72–81; n=4). Germarium 39 (36–42; n=4) width, 44 (35–49; n=4) length. Seminal vesicle 43 (41–47; n=4) width, 43 (42–46; n=4) length. Egg 136 (n=2) length. Haptor 64 (55–68; n=5) width, 65 (58–75; n=5) length. Anchor 61 (59–62; n=5) length; surface bar 6 (5–7; n=3) width, 16 (14–19; n=3) length. Hook 22 (21–24; n=4) length, hook head 5 (4–5; n=4) length.

### Description

Greatest body width at the mid-trunk region; elongated and tapered peduncle. Cephalic lobes and head organs well developed (Fig. 1 A). Testis not observed; spherical seminal vesicles; posterior seminal vesicle thick-walled; seminal vesicle in pre-adults full of spermatozoa, reduced in adults (Fig. 1 C). Male copulatory organ (MCO) muscular, unarmed, copulatory sac with thin wall, delicate vesicle associated with the sinistral branch of the accessory piece. The accessory piece of the MCO is composed of two branches arising from a tubular base, sinistral spatulated branch (Fig. 1 Ca; Ea) and dextral tubular branch (Fig. 1 Cb; Eb). A sinistral spatulated branch composed of two overlapping thin blades bearing subterminal margins with cteniform projections extends beyond the genital pore, guiding the MCO (Fig. 1 Ca; Ea). The dextral branch with median subterminal blade containing cteniform projections and distal region with digitiform projections; articulated, serving as an anchor for the eversion of the MCO and sinistral branch (Fig.1 Cb; Eb; S1. A, B). Germarium subspherical; calyx-shaped uterine pore near the glandular bulb of the pharynx. Egg elongate; filament ¼ the length of the egg; elongated proximal region; bilobed proximal portion of the filament; prominent amorphous cement droplet (Fig. 1 F). Circular haptor. Anchor with an elongated superficial root, short deep root, slightly curved conical shaft and recurved point. Rectangular superficial bar with rounded corners (Fig. 1 G). Hooks similar; hooklet with slightly recurved point, ventrally leaning shaft, rounded heel, and truncated toe; shank maintaining approximately the same width throughout its length, lacks keel; FH loop 1/3 of shank length (Fig. 1 B).

### Remarks

*Aglaiogyrodactylus matildeae* **n. sp.** most closely resembles *A. pedunculatus* in possessing an accessory piece composed of two branches associated with an unarmed tubular copulatory organ. Both species possess a dextral branch with a digitiform termination; however, in *A. matildeae* **n. sp.** this branch bears an expanded subterminal blade provided with cteniform projections, whereas in *A. pedunculatus,* the dextral branch is narrow and lacks such ornamentation. Furthermore, the sinistral branch of *A. matildeae* **n. sp.** is composed of two overlapping blades, the outer blade bearing cteniform projections along its distal margins, whereas the sinistral branch of *A. pedunculatus* is fan-shaped and lacks marginal ornamentation. Additionally, the copulatory complex of *A. matildeae* **n. sp.** is also larger (93 µm) than that of *A. pedunculatus* (75 µm). Hook morphology further distinguishes the two species: *A. pedunculatus* exhibits a more abrupt transition between the shaft and point, whereas *A. matildeae* **n. sp.** presents a smooth and continuous transition between these structures. In addition, the hooks of *A. matildeae* **n. sp.** are markedly shorter (23 µm) than those of *A. pedunculatus* (44 µm) (Kritsky et al., 2007). All remaining congeners can be readily distinguished from *A. matildeae* **n. sp.** by possessing accessory pieces with one, three, or four branches.

**Figure 2.**
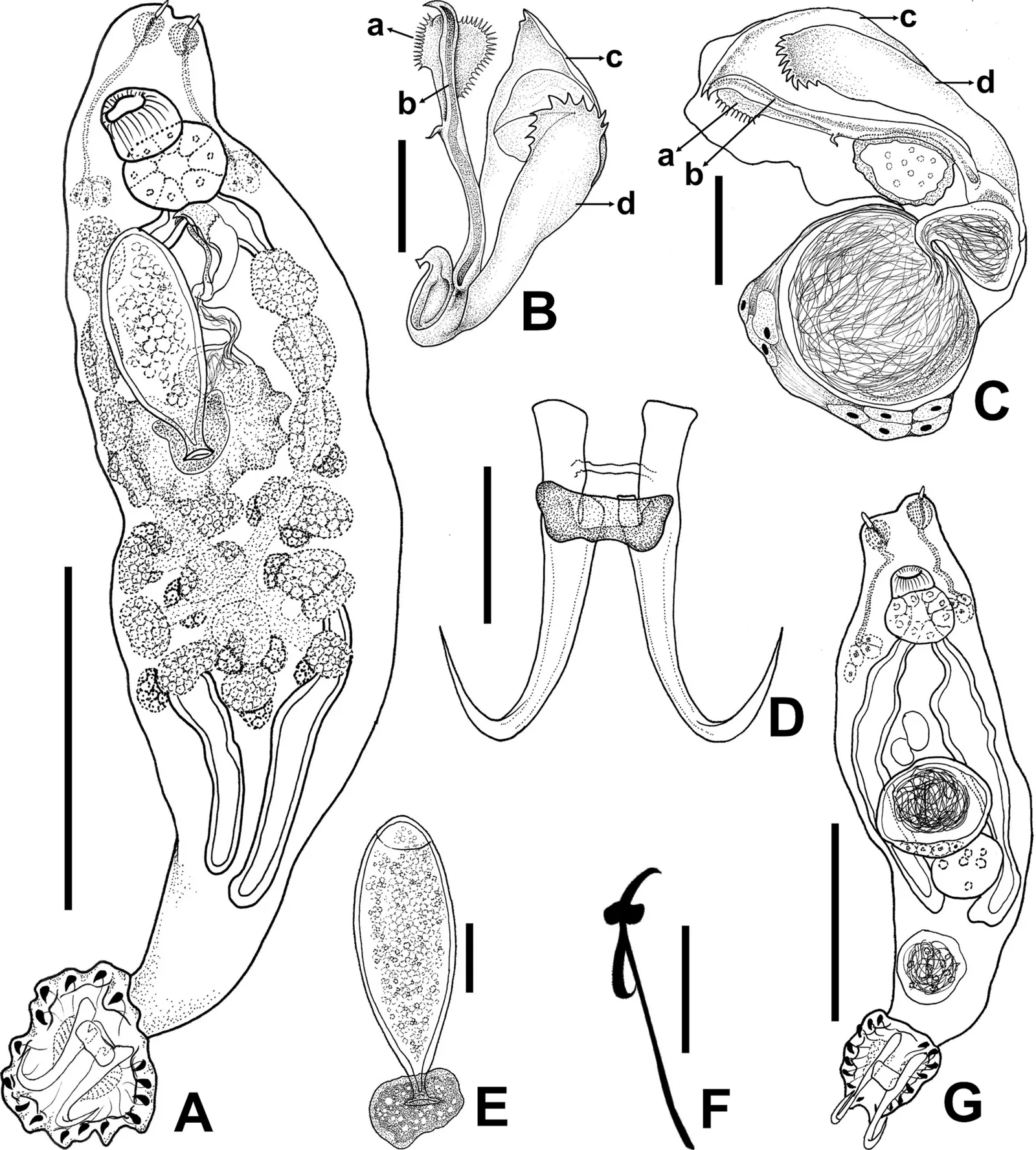
*Aglaiogyrodactylus nonnae* **n. sp. A**: Adult (composite). **B**, **C**: Male copulatory complex; **a** and **b** – dextral branch, **c** and **d** – sinistral branch. **D**: Anchor and bar complex. **E**: Egg. **F**: Hook. **G**: Juvenile. Scale bar: A = 200 µm; B and F = 10 µm; C, D and E = 25 µm; G = 50 µm

***Aglaiogyrodactylus nonnae* n. sp.** (Fig. 2; S1. C, D)

**Host type:** *Hisonotus* sp. (Loricariidae).

**Site of infestation:** Body surface.

**Prevalence:** 65.7% of 38 hosts examined.

**Mean infection intensity:** 5.2 parasites per host.

**Type locality:** Banks of BR 116 (32°08'25.1"S, 52°57'28.6"W), Arroio Grande, Stream near Corredor das Tropas, November 2019.

**Other locations:** Pedro Osório, (31°52'30.7"S, 52°49'06.4"W), Rio Grande do Sul, Brazil, November 2019.

**Specimens deposited:** Holotype, CHIOC 41006; 11 paratypes, CHIOC 41007 – 41017.

**Zoobank:** urn:lsid:zoobank.org:act:E86BC8EF-AF40-416C-BC34-811AA7D4610F

**Etymology:** The specific name is derived from the Italian word "nonna", an affectionate term for grandmothers. This name is a tribute by author Sabrina D. Branchi to "nonnas" Florinda A. G. Dalmas and Iva R. F. Branchi in recognition of their love, dedication, kindness, and strength.

**Measurements**: Body 671 (533–795; n=5) length, 174 (114–215; n=5) width; posterior pharyngeal bulb 54 (46–69; n=3) diameter, anterior pharyngeal bulb 32 (23–40; n=3) diameter. Accessory sinistral piece ‘c’ (Fig. 2Bc) 85 (82–87; n=4), accessory sinistral piece ‘d’ (Fig. 2Bd) 58 (54–62; n=4), accessory dextral piece 68 (65–73; n=4). Germarium 47 (42–51; n=5) width, 55 (50–62; n=5) length. Egg 142 (128–155; n=4) length. Haptor 72 (69–75; n=2) width, 61 (56–66; n=2) length. Anchor 56 (54–58; n=7) length. Surface bar 6 (n=1) width, 16 (n=1) length. Hooks 23 (21–25; n=4) length, hook head 4 (4–5; n=4) length.

### Description

Greatest body width at the germarium; peduncle elongated, maintaining a uniform diameter throughout its length. In adult specimens, the peduncle is elongated, whereas in pre-adults it is shorter, maintaining the proportion according to sexual maturation (Fig. 2A, G). Well-developed cephalic lobes and head organs. Testis not observed in adults, only in juveniles; anterior seminal vesicle oval and thin-walled; posterior seminal vesicle spherical, thick-walled (Fig. 2A, G). Muscular MCO; accessory piece composed of three branches, two sinistral spatulated branches that guide the MCO (Fig. 2Bc, d; Cc, d) and a bifurcated dextral branch (Fig. 2Ba, b; Ca, b). External sinistral spatulated branch with smooth margin and triangular tip (Fig. 2Bc). Inner sinistral spatulated branch with rounded extremity with conspicuous cuneiform projections (Fig. 2Bd). The dextral branch is bifurcated at mid-length, at the level of the thorn-shaped appendage, forming a rounded spatulate branch with a cteniform margin and a second branch with a hooked distal end (Fig. 2Ba, b; S1. C, D). Germarium subspherical; Mehlis gland well developed, lobed. Egg elongate; filament short, with an elongated proximal region and a distally truncated extremity; amorphous cement droplet relatively small (Fig. 2E). Circular haptor; anchor with elongated superficial root, short deep root, both with truncate ends, conical shaft with gentle curvature. Superficial bar in the shape of an inverted trapezoid, with slight central constriction (Fig. 2D). Hooks similar; hooklet with slightly recurved point, ventrally inclined shaft, rounded heel, and upright rounded toe; shank maintaining approximately the same width throughout its length, lacking keel; FH loop length ¼ of shaft length (Fig. 2F).

### Remarks

*Aglaiogyrodactylus nonnae* **n. sp.** most closely resembles *Aglaiogyrodactylus guttus* Kritsky, Vianna & Boeger, 2007 and *Aglaiogyrodactylus salebrosus* Kritsky, Vianna & Boeger, 2007 in possessing an accessory piece composed of three branches. However, *A. guttus* differs from *A. nonnae* **n. sp.** by possessing three terminally acute branches, whereas *A. nonnae* **n. sp.** possesses two overlapping sinistral spatulate branches, one of which bears conspicuous cuneiform projections at its distal extremity. In addition, the accessory piece of *A. nonnae* **n. sp.** is considerably larger (85 µm) than that of *A. guttus* (43 µm). *Aglaiogyrodactylus salebrosus* differs from *A. nonnae* **n. sp.** by possessing three independent branches with ornamented terminations. In contrast, the two sinistral branches of *A. nonnae* **n. sp.** are overlapping, and the dextral branch is bifurcated at mid-length region, with both bifurcations ornamented. The accessory piece of *A. nonnae* **n. sp.** is also larger (85 µm) than that of *A. salebrosus* (64 µm). Although the anchors of these species are morphologically similar, those of *A. nonnae* **n. sp.** are smaller (56 µm) than those of *A. guttus* (65 µm) and *A. salebrosus* (69 µm) (Kritsky et al., 2007). The presence of cuneiform projections on one of the sinistral branches and the morphology of the bifurcated dextral branch readily distinguish *Aglaiogyrodactylus nonnae* **n. sp.** from all other congeners.

**Figure 3.**
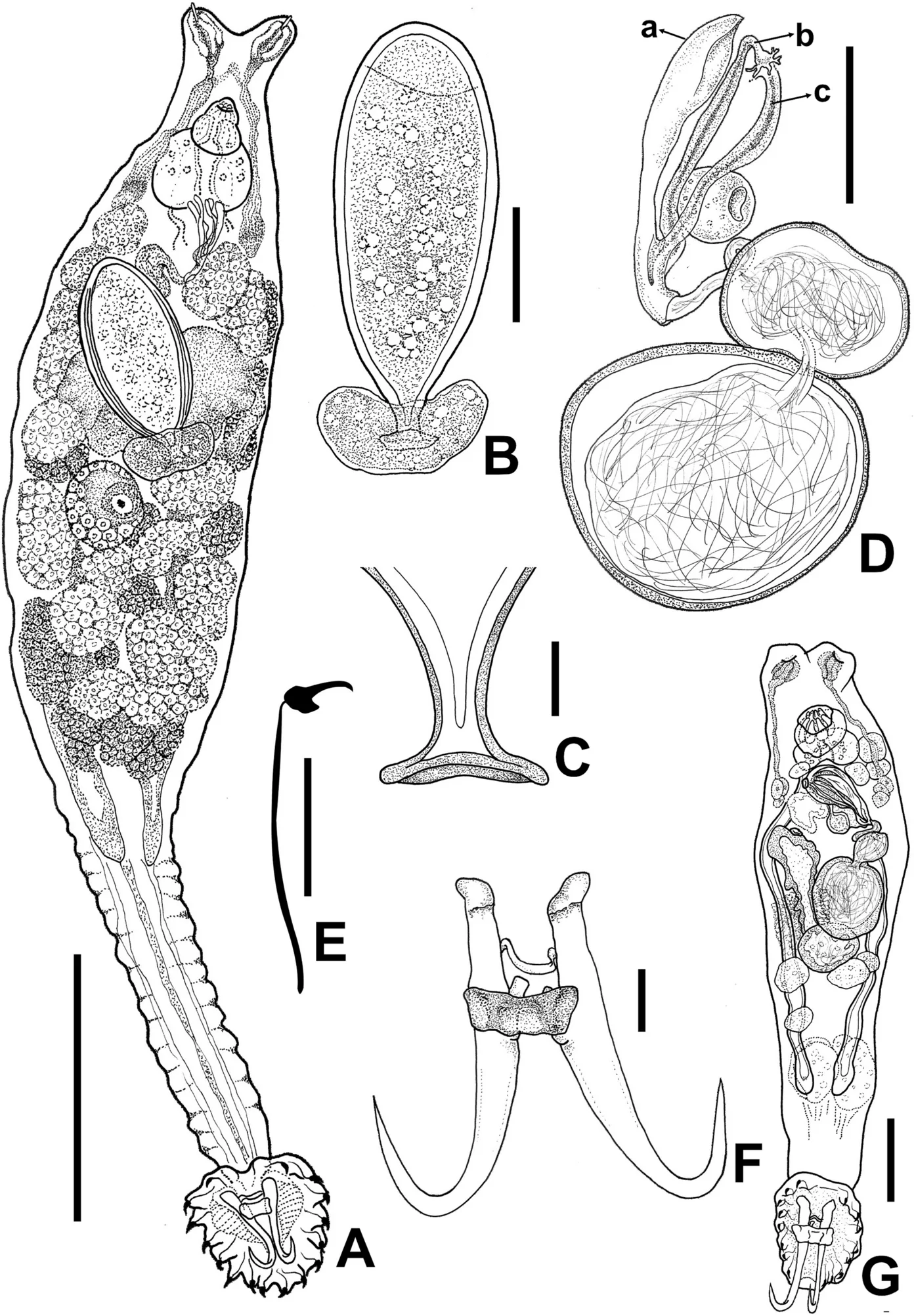
*Aglaiogyrodactylus marinae* **n. sp. A**: Adult. **B**: Egg. **C**: Egg filament. **D**: Male copulatory complex and accessory parts; **a** – dextral branch, **b** and **c** – sinistral branch. **E**: Hook. **F**: Anchor and bar complex. **G**: Juvenile. Scale bars: A = 200 µm; B, D, and G = 10 µm; C and E = 50 µm; F = 25 µm

***Aglaiogyrodactylus marinae* n. sp.** (Fig. 3; S1. E, F)

**Type host**: *Hisonotus* sp. (Loricariidae).

**Site of infestation:** Body surface.

**Prevalence:** 100% of 18 hosts examined.

**Mean infection intensity:** 11.1 parasites per host.

**Type locality:** Stream on the banks of BR 116 (31°08'54.5"S, 52°01'51.1"W), Cristal, Rio Grande do Sul, Brazil, October 2019.

**Other localities:** Banks of BR 116 (31°10'48.5"S, 52°01'32.8"W), Cristal, Rio Grande do Sul, Brazil, October 2019.

**Specimens deposited:** Holotype, CHIOC 40895; 5 paratypes, CHIOC 40896 – 40900.

**Etymology:** The specific name “marinae” is a tribute to Brazilian environmentalist Marina Silva, in recognition of her career and remarkable contribution to biodiversity conservation and protection of the Amazon region.

**Zoobank:** urn:lsid:zoobank.org:act:C2472F1F-4141-43DA-9E68-CF7BAE67713F

**Measurements**: Body 761 (572–1,001; n=10) length, 159 (108–230; n=9) width; posterior pharyngeal bulb 46 (36–56; n=4) diameter, anterior pharyngeal bulb 31 (27–35; n=5) diameter. Accessory piece ‘a’ (Fig. 3Da) 61 (59–64; n=4), accessory piece ‘b’ (Fig. 3Db) 53 (53–57; n=4), accessory piece ‘c’ (Fig. 3Dc) 46 (42–49; n=4). Germarium 47 (32–67; n=5) width, 44 (34–56; n=5) length. Egg 140 (124–165; n=4) length. Haptor 70 (60–87; n=6) width, 65 (55–78; n=8) length. Anchor 59 (55–63; n=10) length. Surface bar 6 (5–7; n=6) width, 17 (16–18; n=6) length. Hooks 22 (20–23; n=4) length, hook head 5 (4–5; n=4) length.

### Description

Greatest body width in the middle region of the trunk; short cephalic region; moderately elongated peduncle. Well-developed cephalic lobes and head organs (Fig. 3A). Testis not observed in adults; in juveniles, the testis overlaps the proximal seminal vesicle; distal seminal vesicle semicircular, thin-walled; proximal seminal vesicle subspherical, thick-walled. Muscular MCO; accessory piece composed of three branches arising from a tubular base; spatulated sinistral branch with a semicircular and grooved distal end; stick-shaped dextral branches bearing inconspicuous digitiform projections distally (Fig. 3Da, c; S1. E, F). Germarium subspherical. Egg elongated; eggshell multilayered; filament short, with broad and calyx-shaped proximal region; amorphous cement droplet, prominent, compressed anteroposteriorly, forming a concavity on the anterior surface (Fig. 3B, C). Circular haptor. Anchor with elongated superficial root, short deep root with straight end, slightly curved conical shaft with gentle curvature. Superficial bar in the shape of an inverted trapezoid, with rounded and thickened lateral ends; deep bar in the shape of a stick (Fig. 3F). Hooks similar; hooklet with slightly recurved point, ventrally inclined shaft, rounded heel, and upright toe; shank tapering distally toward the point, lacking keel; FH loop length ¼ of shaft length (Fig. 3E).

### Remarks

*Aglaiogyrodactylus marinae* **n. sp.** most closely resembles *A. guttus* and *A. salebrosus* in possessing an accessory piece composed of three branches. *Aglaiogyrodactylus guttus* differs from *A. marinae* **n. sp.** by possessing three terminally acute branches, whereas *A. marinae* **n. sp.** possesses a spatulate sinistral branch with a semicircular grooved distal extremity and two stick-shaped dextral branches. In addition, the accessory piece of *A. marinae* **n. sp.** is larger (61 µm) than that of *A. guttus* (43 µm). Although the anchors of both species are morphologically similar, those of *A. marinae* **n. sp.** are smaller (59 µm) than those of *A. guttus* (65 µm). The hooks of *A. marinae* **n. sp.** are also considerably shorter (22 µm) than those of *A. guttus* (40 µm), whose hooklets possess a more acute point (Kritsky et al., 2007).

*Aglaiogyrodactylus salebrosus* also differs from *A. marinae* **n. sp.** markedly in the morphology of the accessory piece. Although the accessory pieces of *A. marinae* **n. sp.** and *A. salebrosus* are similar in length, 61 µm and 64 µm, respectively. In *A. salebrosus*, the dextral branch bears a cteniform distal termination and both sinistral branches possess digitiform terminations. In contrast, in *A. marinae* **n. sp.**, the accessory piece comprises a spatulate sinistral branch with a semicircular grooved distal extremity and two stick-shaped dextral branches bearing inconspicuous digitiform projections distally. Although the hooks of both species are morphologically similar, those of *A. marinae* **n. sp.** are markedly shorter (22 µm) than those of *A. salebrosus* (46 µm). The anchors of *A. marinae* **n. sp.** are also smaller (59 µm) than those of *A. salebrosus* (69 µm). All remaining congeners can be readily distinguished from *A. marinae* **n. sp.** by possessing accessory pieces with one, two, or four branches (Kritsky et al., 2007).

***Aglaiogyrodactylus pedunculatus*** **Kritsky, Vianna & Boeger, 2007**

**Type host:** *Hisonotus* sp. (Kritsky et al., 2007)

**Other hosts:** *Hisonotus leucofrenatus* (Miranda Ribeiro), *Pareiorhaphis splendens* (Bizerril), *Peckoltia multispinis* (Holly) *Rineloricaria* sp., *Schizolecis guntheri* (Miranda Ribeiro) (Patella et al., 2017)

**New host record**: *Hisonotus* sp. (Loricariidae)

**Site of infestation:** Body surface.

**Type location:** Dois de Fevereiro River (25°26’S, 48°42’W), Antonina, Paraná State, Brazil (Kritsky et al., 2007)

**Other localities:** Marumbi River (25°30’32.8” S, 48°52’31.3” W), Paraná state (Patella et al., 2017)

**New location record:** Stream on the banks of BR 116 (31°10'48.5"S, 52°01'32.8"W), Cristal, Rio Grande do Sul state, Brazil, October 2019.

### Remarks

The morphological characteristics observed in the specimens examined herein agree with the diagnosis of *Aglaiogyrodactylus pedunculatus* provided by Kritsky et al. (2007). A comparison between the measurements reported in the original description and those obtained in the present study is presented in Table 2. Except for the superficial bar and hook measurements, the ranges of all examined structures overlapped with those reported by Kritsky et al. (2007). Specimens examined in the present study exhibited a smaller superficial bar (17 µm; range 16–18 µm, n = 5) than that of the type series (24 µm; range 20–29 µm, n = 14). Likewise, hook shank lengths were smaller in the present material (22 µm; range 20–23 µm, n = 4) than in the type series (44 µm; range 41–47 µm, n = 27) (Table 2). However, the dimensions of the hooklet point, a structure considered to be of greater taxonomic importance for species discrimination, were consistent with those reported in the original description. *Aglaiogyrodactylus pedunculatus* was found co-occurring with *A. marinae* **n. sp.**

**Table 2:** Comparative morphometric data for *Aglaiogyrodactylus pedunculatus* Kritsky, Vianna & Boeger, 2007 from the original description of Kritsky et al. (2007) and the material examined in the present study

Structure		Present study	Kritsky et al. (2007)
Body	Width	151 (108–181) n=5	140 (100–209) n=31
	Length	748 (572–1,001) n= 6	1,006 (615–1,455) n=32
Pharynx	Posterior bulb	46 (36–56) n= 3	55 (44–69) n=30
	Anterior bulb	31 (31–32) n= 3	37 (32–42) n=30
Germarium	Length	140 (124–165) n= 3	162 (150–172) n=16
Haptor	Width	72 (60–87) n=4	95 (71–147) n=29
	Length	68 (58–78) n=5	103 (81–121) n=19
Anchor	Width	7 (6–8) n=6	–
	Length	59 (55–63) n= 6	69 (62–78) n=25
Surface bar	Width	6 (5–7) n=5	–
	Length	17 (16–18) n=5	24 (20–29) n=14
Hook	Length	22 (20–23) n=4	44 (41–47) n=27
	Hook head	6 (5–7) n=4	6 (6–7) n=15

## Discussion

Since the establishment of the Oogyrodactylidae by Harris (1983), approximately twenty species have been described parasitizing siluriform hosts, particularly within the Loricariidae and Pimelodidae Swainson. Nevertheless, the diversity of oogyrodactylids is likely underestimated, as less than 4% of the potential host diversity has been examined for the presence of these parasites (Branches et al., 2021). The limited number of parasitological surveys focusing on loricariid and pimelodid hosts suggests that a significant portion of oogyrodactylid diversity remains undiscovered. In the present study, three new species of *Aglaiogyrodactylus* are described, and a previously described species is reported for the first time from *Hisonotus* spp. from Rio Grande do Sul State, Brazil. These findings expand the known geographical range and morphological diversity of Oogyrodactylidae, providing further evidence of the diversification of this family among Neotropical loricariid fishes.

Oogyrodactylidae was proposed by Harris (1983) to accommodate two oviparous species and was later substantially expanded by Kritsky et al. (2007), who described several new species and genera, including *Aglaiogyrodactylus*. Over the past two decades, no additional species of the genus have been described, and subsequent contributions have been limited to new host and locality records (Patella et al., 2017). Thus, this study represents an important contribution to the knowledge of *Aglaiogyrodactylus* diversity, increasing the number of recognized species by 37.5% (from 8 to 11). Comparative analyses reveal a complex pattern of morphological differentiation among the species described by Kritsky et al. (2007) and those proposed herein. Species delimitation is supported primarily by variation in the morphology of the male copulatory complex, whose consistent interspecific differentiation highlights its value as a diagnostic character. Collectively, these findings reinforce the distinctiveness of the species recognized within *Aglaiogyrodactylus* and highlight the remarkable morphological complexity observed among oogyrodactylids.

More specifically, a comparative analysis of the species proposed herein highlights the importance of the morphology of the accessory pieces of the male copulatory complex in distinguishing among *Aglaiogyrodactylus* species. *Aglaiogyrodactylus matildeae* **n. sp.** and *Aglaiogyrodactylus nonnae* **n. sp.** exhibit accessory pieces with laminar morphology, a condition previously recorded only in *A. pedunculatus* (Kritsky et al., 2007). This structural similarity contrasts with the morphological diversity observed among the remaining congeners, suggesting that the laminar condition may represent a potentially informative morphological character for systematic comparisons within *Aglaiogyrodactylus*.

All *Aglaiogyrodactylus* species with laminar accessory pieces have been described from *Hisonotus* hosts (Kritsky et al., 2007). While species of this genus also occur on other loricariids (Table 1), the recurring presence of this morphological pattern in those associated with *Hisonotus* hosts is a notable feature. Nevertheless, the biological significance of this pattern remains unclear. The presence of laminar accessory pieces in *A. pedunculatus*, *A. matildeae* **n. sp.**, and *A. nonnae* **n. sp.** highlights a morphological feature useful for systematic comparisons within *Aglaiogyrodactylus*. However, morphology alone is insufficient to assess its evolutionary significance; therefore, future studies integrating molecular data and detailed morphological analyses will be necessary to determine whether these similarities result from common ancestry, convergent evolution, or other evolutionary processes within oogyrodactylids.

The observed co-occurrence of *A. pedunculatus* and *A. marinae* **n. sp.** is consistent with previous records of congeneric species infecting the same host. Patella et al. (2017) reported *A. pedunculatus* co-occurring with other *Aglaiogyrodactylus* species on loricariid hosts, including *A. calamus*, *A. forficulatus*, and *A. guttus*. Furthermore, their study demonstrated that individual loricariid hosts may harbor multiple species of *Aglaiogyrodactylus* simultaneously, with up to five congeneric species recorded from a single host species. The present findings provide an additional record of co-occurrence and further support previous observations that multiple congeners may coexist on the same loricariid host.

The present study expands the known diversity of *Aglaiogyrodactylus* through the description of three new species from loricariid hosts in the Neotropical Region and by reporting *A. pedunculatus* from Rio Grande do Sul, Brazil. These findings increase the number of recognized species within the genus and broaden current knowledge of its host associations and morphological diversity. The results also highlight the importance of taxonomic surveys in Neotropical freshwater systems, where oogyrodactylid diversity remains poorly documented. Continued sampling of loricariid hosts across different hydrographic basins will likely reveal additional species and contribute to a more comprehensive understanding of the distribution and diversity of these parasites.

## Supplementary Information

Below is the link to the electronic supplementary material.

Supplementary file1 (DOCX 2495 kb)

## Data Availability

No datasets were generated or analysed during the current study.
