# Supplementary material for: Three New Species and a New Record of Aglaiogyrodactylus (Monopisthocotyla, Gyrodactylidea, Oogyrodactylidae) from Hisonotus spp. (Siluriformes, Loricariidae) in Brazil
Source: Syst Parasitol. 2026 Aug 3;103(5):35. doi: 10.1007/s11230-026-10293-9 (PMC13433391; doi:10.1007/s11230-026-10293-9)
Supplement: Supplementary file 1 [file 11230_2026_10293_MOESM1_ESM.docx]

**Supplementary material 1.** Male copulatory complexes of the three new species of *Aglaiogyrodactylus* Kritsky, Vianna & Boeger, 2007. **A–B**: *Aglaiogyrodactylus matildeae* **n. sp.**, with (**a**) the sinistral branch and (**b**) the dextral branch. **C–D**: *Aglaiogyrodactylus nonnae* **n. sp.**, with (**a, b**) the dextral branch and (**c, d**) the sinistral branch. **E–F**: *Aglaiogyrodactylus marinae* **n. sp.**, with (**a**) the dextral branch and (**b, c**) the sinistral branch.
